# Resident Memory T Cells in Autoimmune Skin Diseases

**DOI:** 10.3389/fimmu.2021.652191

**Published:** 2021-05-03

**Authors:** Grace E. Ryan, John E. Harris, Jillian M. Richmond

**Affiliations:** Department of Dermatology, University of Massachusetts Medical School, Worcester, MA, United States

**Keywords:** resident memory T cell (TRM), cutaneous lupus erythematosus (CLE), vitiligo, psoriasis, alopecia, dermatology, autoimmunity

## Abstract

Tissue resident memory T cells (TRM) are a critical component of the immune system, providing the body with an immediate and highly specific response against pathogens re-infecting peripheral tissues. More recently, however, it has been demonstrated that TRM cells also form during autoimmunity. TRM mediated autoimmune diseases are particularly destructive, because unlike foreign antigens, the self-antigens are never cleared, continuously activating self-reactive TRM T cells. In this article, we will focus on how TRMs mediate disease in autoimmune skin conditions, specifically vitiligo, psoriasis, cutaneous lupus erythematosus, alopecia areata and frontal fibrosing alopecia.

## Introduction

Tissue resident memory T cells (TRM) are long-lived lymphocytes that reside in tissues and develop after a T cell-mediated immune response is initiated. Subpopulations of memory T cells can recirculate through the blood and lymphoid organs T recirculating memory cells (TRCM), though evidence suggests that true TRM have a reduced capacity for recirculation. Both CD4+ and CD8+ TRM have been described in skin and other tissues including the mucosa, lung, brain, GI tract and pancreas (1–5). Because of the very different locations that TRM are found, they are an extremely diverse population, showing specializations for their resident tissue. Mouse models reveal that TRMs function as sentinel-alarm cells that secrete cytokines and chemokines upon encountering their cognate antigen (6–8), with some infections such as vaccinia virus requiring TRM for optimal viral clearance (9). There is mounting evidence that TRM work together with other effector and memory T cell populations to provide tissue surveillance and clear infections (8, 10).

**Figure 1:**
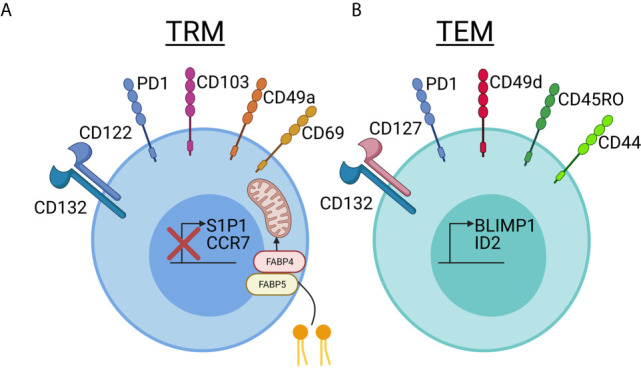
Comparing and contrasting tissue resident memory and effector memory T cell phenotypes in skin. **(A)** Tissue resident memory (TRM, left, blue) cells downregulate S1P1 and CCR7, which are used for recirculation in the blood and lymph, when they take up residency in skin. TRMs use CD69, which was previously thought to be a marker of recently activated T cells, to block the expression of S1P1 on the cell surface and prevent exit from the tissue. CD103 is an integrin family member that is involved in skin and mucosal homing. It is not required for skin retention, but is expressed at a much higher rate in TRM cells in the epidermis compared to T cells in the dermis. CD49a has recently been used to distinguish the CD8+CD103+CD49a+ TRM T cells involved in mediating disease in vitiligo from the CD8+CD103+CD49a- TRM cells involved in psoriasis. TRM cells survive long-term in the nutrient-poor conditions of the skin by metabolizing free fatty acids. Increased expression of FABP4 and FABP5, fatty-acid binding proteins, allows TRM cells to take up larger amounts of exogenous free fatty acids for energy. IL-15 is required for TRM differentiation in skin, and TRM bear the CD122 and CD132 receptor chains. Keratinocytes can present IL-15 to TRM in trans on the CD215 receptor chain. TRM cells increase expression of PD1, which has a known inhibitory role in immune activation, and is important in maintaining immune homeostasis. Reduced expression of PD-1 in TRMs is associated with inappropriate chronic inflammation. CD69, which was historically thought to be a T cell activation marker, is stably expressed on Trm. **(B)** T effector memory (TEM, right, green) cells can also express varying levels of PD-1, making this marker useful more for determining exhaustion and/or chronic inflammation rather than tissue residence. TEM seem to rely more on CD127/IL-7 as a survival factor, which is in contrast to the IL-15 dependency of TRM. More often, they express CD49d than CD49a, though CD49a may also be used as a marker of degranulation. CD49d can interact intracellularly with CD44 to promote signaling, though CD44 is often present on both TRM and TEM and is used as a marker of “antigen experienced” T cells. CD45RO has historically been used to identify TEM in blood, and this short isoform of CD45 plays a role in T cell activation and signaling though a variety of pathways including JAK/STAT and Src. The transcription factors BLMP1 and ID2 promote expression of TEM-associated markers. Created with BioRender.com.

Compared to TRM in other tissues, skin TRM persist for years and express skin-specific homing antigens, such as cutaneous lymphocyte antigen (CLA) and CCR8. In addition, certain skin retention markers, including CD103 and CD69, are upregulated (11). Mouse models have demonstrated that TRM require IL-15 and TGFβ for their differentiation in the skin (12), and human TRM express their receptors (3, 13). The tissue-residency program that ensues involves downregulation of chemokine receptors involved in recirculation, including CCR7 and S1P1, as well as upregulation of key integrins, cytokine/growth factor receptors, and signaling molecules including CD69, CD103, CD49a, CD122 and PD-1, a pattern that is conserved in both mice and humans (5, 14–16). Skin TRM survival is dependent upon IL-15 and fatty acid metabolism (3, 17) (**Figure 1**).

In addition to anti-viral responses, TRM also develop in the context of xenobiotic and/or toxin exposure, allergies and autoimmune diseases. For example, fixed drug eruption is thought to be mediated by TRM (18). Type IV hypersensitivity to poison ivy, other contact dermatitis irritants or allergens induces TRM development (19). Healthy skin donors exhibit autoreactive T cell clones, which are thought to provide immune surveillance against tumors (3, 20). However, in autoimmune skin conditions, environmental and genetic factors precipitate inappropriate activation of autoreactive T cells and promote onset of autoimmunity. Further, a reduced ratio of Tregs to TRM and T effectors and/or reduced functionality of Tregs in skin tissue tips the balance towards development of autoimmunity (21–25), which may become recalcitrant or relapse following treatment cessation due to the persistence of autoreactive TRM. In this minireview, we will focus on TRM in autoimmune skin diseases, including discussions of how they may be targeted for durable treatment options.

## TRM in Vitiligo

Vitiligo is characterized by distinct patchy white spots present on patients’ skin, which are caused by CD8+ T cells inappropriately targeting melanocytes for destruction. Many studies have implicated both genetic and environmental factors are important in the pathogenesis of vitiligo (26). Familial clustering of vitiligo suggested that there was a genetic component before genetic studies were available. Alkhateeb et al. reported in 2003 that approximately 20% of people with vitiligo reported at least one affected first-degree relative (27). Since then, genome-wide association studies (GWAS) have identified multiple genes associated with a higher risk for developing vitiligo, including genes involved in both innate and adaptive immunity (28, 29). It is now recognized that melanocytes in vitiligo patients have a reduced ability to manage cellular stress, making them more vulnerable to environmental stressors such as UV radiation and certain chemicals (30, 31). Cytokines and danger associated molecules released in response to states of high stress in the skin attract and activate immune cells (32–34). In addition, the IFNγ-CXCR3 pathway has been determined to be critical to the migration of autoreactive CD8+ T cells in vitiligo in mice and humans, and correlates with disease progression and severity (35–39). While treatments are available for vitiligo patients, the white spots rapidly reappear in the same location if therapy is stopped, demonstrating that memory persists in the skin even after treatment. For example, JAK/STAT signaling is recognized as a key mediator in many of the effector functions of TRM cells, including the production of cytokines and inflammation inducers. JAK inhibitors interrupt the IFNγ signaling pathway and cause skin repigmentation; however, depigmentation recurs when treatment is stopped (40). Azzolino et al. recently reported that established TRM numbers in skin in a mouse model of vitiligo are not affected by Jak inhibitors, thus providing an explanation for why JAKi do not provide durable treatment responses (41).

T cells in vitiligo patients react to melanocyte self-antigens including gp100/Pmel-17, melan-A/MART-1, tyrosinase and tyrosinase-related proteins 1 & 2 (42–47). Recent studies have characterized melanocyte-specific CD8+ TRMs from patients with active and stable vitiligo using blister biopsies and punch biopsies (3, 4, 48). Approximately 80% of the melanocyte-specific CD8+ T cells express TRM markers, including CD69 single positive cells (which may be actively differentiating, or recirculating cells) and CD69/CD103 double positive cells (3). CD49a, a marker that distinguishes a subset of CD8+ CD103+ T cells with the cytotoxic potential to produce IFNγ, is expressed in half of epidermal and less than a quarter of dermal CD8+ CD103+ T cells in patients with vitiligo. Comparatively, in healthy skin, it is expressed by one third of epidermal and less than 5% of dermal CD8+ CD103+ T cells (4). In all patients with vitiligo, these cells were enriched in the skin; moreover, in patients with active disease, there was a significant increase in the number of T cells as compared to those with stable disease (36), particularly CD69+ (3). Boniface et al. found co-expression of CXCR3 on CD69+CD103+ melanocyte-specific T cells in vitiligo skin at a higher frequency than psoriasis or healthy skin (48). CD8+ TRM are also present in depigmenting lesions in post-melanoma-immunotherapy-associated vitiligo (49).

**Figure 2:**
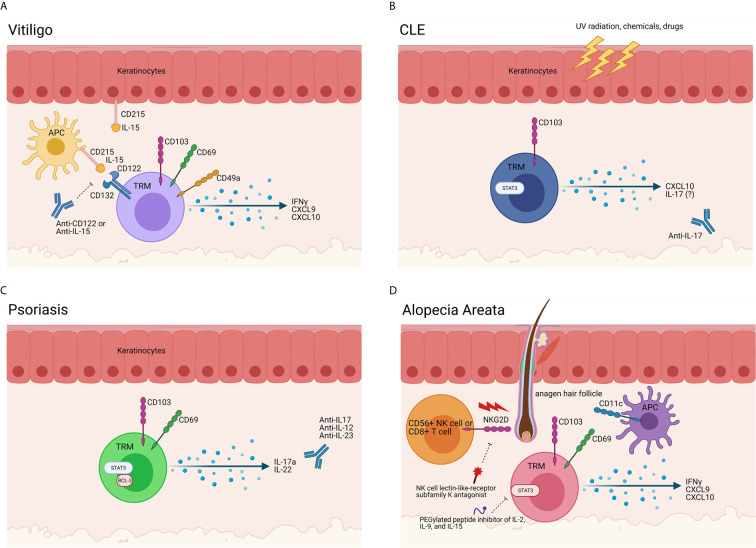
Evidence of TRM in autoimmune skin disease. **(A)** TRM in vitiligo. Melanocyte-specific TRM are present in vitiligo lesional skin. These cells express CD49a, CD69, CD103, and CD122 and produce IFNγ and chemokines upon encountering cognate antigen, such as gp100/Pmel-17, melan-A/MART-1, tyrosinase and/or tyrosinase-related proteins 1 & 2 presented on HLA-A2. Antigen-presenting cells (APCs) and keratinocytes also play a role in vitiligo immunopathogenesis, as they release cytokines in response to a myriad of stimuli and stressors. APCs produce IL-15 and trans-present it on CD215 to T cells, which bear the CD122 and CD132 chains of the IL-15 receptor. In vitiligo, our data support that TRM act as sentinel/alarm cells to induce recall responses against melanocytes. Blocking CD122 deprives the TRM of the IL-15 survival signal, thereby inhibiting their function and/or reducing their numbers to provide a durable treatment. A clinical trial is currently enrolling to test the potential of a commercially available anti-IL-15 antibody for vitiligo (NCT04338581). **(B)** TRM cells in CLE. Triggers such as UV radiation, infection, hormones, drugs and chemicals create an apoptotic and inflammatory environment (50). In lupus-prone individuals, antigen-presenting cells (APCs) migrate to the site of injury and ingest the cell debris and possible autoantigens. When the APCs present the autoantigens to self-reactive T cells and B cells, the autoimmune response is initiated, and disease occurs (51). Specifically, CD103+ TRM cells in the skin are positively correlated to disease severity and recurrence in patients. While TRM and T cell antigen specificities in lupus nephritis were recently described (52, 53), specificities of skin autoreactive T cells are unknown. A trial blocking anti-IL-17 antibody for DLE is recruiting (NCT03866317). **(C)** TRM cells in Psoriasis. CD69+ CD103+ TRM are present in psoriasis lesions. The production of cytokines such as IL-17a, IL-22, and Bcl-3 by these TRM cells lead to aberrant keratinocyte proliferation and differentiation. A recent study described melanocyte-specific T cells in psoriasis patients, though other specificities are unknown (54). Many trials for IL-17 family cytokine blockade, including IL-12/23, are ongoing. **(D)** TRM cells in Alopecia Areata (AA) and Cicatricial Alopecia (CA). NKG2D+ CD8+ T cells begin the disease process, breaching the immune privileged hair follicle through the activation of T cells and dendritic cells. Though the exact role of TRM cells in the pathogenesis of AA and FFA is unknown, it is clear that the production of IFNγ, CXCL9, and CXCL10 by T cells in the hair follicle is crucial in the development in disease. While clonal expansions of CD8 T cells have been detected with TCR sequencing (55), the antigen specificities of TRM in alopecias are still largely unknown (56). Trials for NK cell receptor and IL-2/9/15 inhibition are planned (NCT03532958 and NCT04740970). Created with BioRender.com.

We examined factors required for maintenance of these autoreactive TRM in the skin during vitiligo, with the hope of identifying new durable treatments targeting autoreactive TRM. Based on Mackay et al, which demonstrated TRM require IL-15 for their differentiation, we tested whether blocking this cytokine pathway could deplete TRM. Treating mice with anti-CD122 antibodies, which block IL-15 signaling and thus TRM proliferation and survival, provided long-lasting repigmentation (8) (**Figure 2A**). Other labs have mapped additional roles IL-15 plays in vitiligo. Chen et al. demonstrated that oxidative stress can result in increased IL-15 transpresentation on CD215 by keratinocytes (34). Jacquemin et al. demonstrated that IL-15 can induce NKG2D expression on effector memory CD8+ T cells (57), which, based on our FTY720 blockade data demonstrating that TRM are not sufficient for maintaining depigmentation in mice (8), supports a role for effector memory T cells as the melanocyte killers. IL-15 blocking antibodies are now being explored as a potential durable therapy for vitiligo patients (NCT04338581; **Table S1**).

## TRM in Cutaneous Lupus Erythematosus

Cutaneous lupus erythematosus (CLE) is a spectrum of autoimmune skin diseases encompassing several clinical subtypes, all of which are characterized by interface dermatitis, or inflammation at the dermal-epidermal junction (58). The most well-known manifestation of CLE is the characteristic erythematous malar rash, which is associated with systemic lupus erythematosus (SLE). Other CLE subtypes can present without any systemic involvement, with different entities conferring different levels of risk of developing SLE. Genetic factors and environmental stressors both contribute to the etiology of CLE (**Figure 2B**).

The T cell specificities in CLE skin are largely unknown, but SLE studies identified T cells reactive to nucleosomes/histones (59), which can induce anti-dsDNA antibody production (60). A recent study characterized antigen specificities of T cells in the kidney in lupus nephritis patients, and found that they recognize SmD1, RNP70, histone, Ro, La, and nuclear antigen (52, 61). A population of CD8+ CD103+ tissue-resident memory T cells was identified and correlated positively with disease severity in patients (53). Interestingly, tofacitinib, a JAK3>1>>2 inhibitor, was effective in preventing disease progression in mouse models of lupus nephritis and suppressed the development of CD8+ TRM cells. Thus, the microenvironment that the TRM cells create in lupus nephritis appears to be crucial for disease progression.

Many studies have shown aberrant T cell signaling contributes to the pathogenesis of CLE (62), including expression of cytotoxic markers characteristic of T cell function (63, 64). However, it is only recently that TRM cells have been linked to disease progression: In the skin, increased TRM were found in CLE patients refractory to antimalarials (65). JAK/STAT inhibitors have also demonstrated efficacy in treating CLE: In a case study of a patient with chilblain lupus, a subtype of CLE, the JAK 1/2-kinase inhibitor ruxolitinib provided a rapid response. When ruxolitinib was discontinued, the skin lesions re-appeared, but upon re-starting the drug they resolved completely (66). Thus, similar to vitiligo studies, TRM in CLE are not affected permanently by Jak inhibitors, and disease recurs once treatment is stopped. Examination of skin biopsies taken from the patient revealed that ruxolitinib inhibited the production of CLE-typical chemokines, specifically CXCL10, supporting the idea that TRM cytokine production and downstream signaling drives recruitment of recirculating cytotoxic cells to the skin, as was demonstrated in vitiligo mouse models described above. Another study identified IL-17+ cells by immunohistochemistry (67); a trial assessing IL-17 blockade for discoid lupus erythematosus is recruiting (**Table S1**).

## TRM in Psoriasis

Psoriasis is a chronic and relapsing inflammatory skin disease mediated by T cells. As in other autoimmune skin diseases, the relapsing nature of psoriasis suggested the presence of immune memory in the skin. Using a humanized mouse model of psoriasis, transplantation of unaffected skin from a patient with psoriasis was grafted onto mice deficient in type I and type II interferons and the recombination activating gene 2, (IFNG-/- IFNAR-/- RAG2-/-) and the skin graft spontaneously developed psoriasis. Thus, the immune cells already resident in the grafted skin were sufficient for inducing disease (68). Moreover, E-selectin inhibitors are not efficacious in preventing psoriasis, further supporting that nonmigratory immune cells can mediate disease (69). However, the question of whether TRM are sufficient, or whether they also require TRCM, has not yet definitively been addressed, because the resident immune cells in skin graft experiments may have given rise to TRCM, and there may be redundant pathways in addition to E-selectin that mediate skin recruitment in psoriasis.

Cheuk et al. demonstrated that the marker CD49a identifies two distinct populations of CD8+ CD103+ TRM cells. While vitiligo disease is driven by CD49a+ TRM cells producing IFNγ, CD49a- TRM cells in psoriasis mediate disease through the production of interleukin-17 (IL-17) (4, 70), (**Figure 2C**). The identification of CD49a- IL-17+ T cells as critical mediators of disease fits with the previous success of anti-IL-17 drugs that mediate successful amelioration of disease (UptoDate). The FDA has approved several monoclonal antibodies to IL-17 for the treatment of psoriasis: tildrakizumab, guselkumab, risankizumab, secukinumab, ixekizumab, and brodalamub (**Table S1**). Scientists have also developed drugs to target IL-23, a cytokine produced by myeloid dendritic cells to activate IL-17-producing CD49a- TRM cells as well as non-TRM IL-17-producing cells (71). Nevertheless, IL-17 blockade may require long-term administration, as it is blocking a molecule made by the TRM rather than targeting TRM themselves. In a clinical study of ten patients with psoriasis receiving secukinumab for 24 weeks, Fujiyama et al. demonstrated that while there was a significant decrease in CD8+ CD103+ cells in lesional skin, there was only a slight decrease in CD8+ CD103+ CD49- cells, suggesting that TRM cells are preserved (72).

While CD8+ CD103+ TRM cells make up the majority of the T cell infiltrate in psoriatic lesions, CD4+ TRM cells are also present in low numbers in the epidermis and dermis (73). Like CD8+ TRM, CD4+ TRM are identified by the marker CD69+. However, unlike CD8+ TRM, CD4+ TRM cells show variability in the expression of CD103, and the majority of CD4+ TRM do not express it. CD4+ TRM were further distinguished from CD8+ TRM cells by Cheuk et al, who demonstrated that epidermal CD4+ TRM cells in resolved psoriatic lesions produced IL-22 while CD8+ T cells mainly produced IL-17A. IL-22 is a pro-inflammatory cytokine that promotes the expression of multiple chemokines in the skin (74). In addition, through the STAT3 pathway, IL-22 promotes the proliferation and decreases the differentiation of keratinocytes, a hallmark of psoriatic skin (75). Using immunohistochemistry, Tohyama et al. reported that the production of IL-17A by CD8+ TRM cells and IL-22 by CD4+ TRM cells correlated with Bcl-3 production, IL-22-induced gene expression, and expression of other genes associated with psoriasis in lesional skin as compared to healthy control skin. By targeting Bcl-3, both CD4+ and CD8+ TRM disease pathways in psoriasis could be interrupted (76). It is now understood that expression of Bcl-3 promotes CD4+ T cell survival and is necessary for the proper development of Th1, Th2, and Th17 cells (77). While Bcl-3 inhibitors have been extensively studied as potential therapeutics in cancer, their role as a therapeutic in psoriasis or other skin diseases has not yet been explored.

Although blocking the psoriatic microenvironment produced by TRM cells has proven effective in treating disease, scientists are also looking at how to directly target TRM cells. Pan et al. examined the metabolic pathways of mouse CD8+ TRM cells in order to elucidate how TRM cells survive in the nutrient-poor conditions of the skin. In both mouse models and skin biopsies from patients with psoriatic lesions, it was found that TRM T cells required exogenous free fatty acids to survive in the skin. This characteristic is unique to TRM T cells and could be a potential target for treatment for skin diseases mediated by TRM T cells (17).

## TRM in Alopecia Areata and Cicatricial Alopecia

The hair follicle has long been recognized as an immune privileged site, as it protects the hair from potential autoreactive immune attacks through a system of complex regulation and suppression of the immune system. When this system breaks down, hair loss can result (78–80). Alopecia areata (AA) and cicatricial alopecia (CA) are common, inflammatory hair loss conditions. While CA is scarring and has a distinct clinical presentation of hair loss along the frontotemporal hairline and eyebrows, AA can present variably, ranging from a single patch of hair loss to total body hair loss and is non-scarring (56, 81). A combination of genetics and environmental triggers have been implicated in the pathogenesis of both alopecias.

CA is a diverse group of scarring hair loss encompassing several clinical variants including frontal fibrosing alopecia (FFA), lichen planopilaris (LPP) and central centrifugal cicatricial alopecia (CCCA). FFA and LPP most commonly occur in postmenopausal Caucasian women, however there have been a few cases in younger women (82). Autoimmune thyroid disease is the most common comorbid autoimmune disorder with FFA and LPP. In contrast, CCCA almost exclusively occurs in African American women (83), and Type 2 diabetes is the most common comorbidity.

AA affects people of all different ages, sexes, and ethnic backgrounds (84). To date, the immunopathogenesis of AA is the most well-characterized of the alopecias: AA is driven by CD8+ T cells and natural killer (NK) cells as evidenced by both mouse models and studies of ex vivo human tissues (80). NKG2D+ cells are central to pathogenesis (85), as NKG2D activates both CD8+ T cells and NK cells to promote destruction of the hair follicle. A clinical trial targeting NK cell lectin-like-receptor subfamily K is currently enrolling (**Table S1**).

While anagen hair follicle epithelium is the target of CD8+ T cells in AA, FFA is mediated by destruction of stem cells in the hair follicle (86). Targets in CCCA have not been well-characterized, and future studies of alopecia in patients with skin of color are warranted to understand nuances in immunopathogenesis and potential novel treatment options.

Del Duca et al. recently determined that lesional skin from AA and FFA patients has significantly more CD8+ cytotoxic T cells, CD11c+ dendritic cells, and CD103+ CD69+ TRM cells when compared to nonlesional skin (87). In particular, FFA was shown to have a significant upregulation of the IFNγ/CXCL9/CXCL10 pathway and the JAK-STAT pathway (88), (**Figure 2D**). In a recent case series, Yang et al. reported significant clinical improvement as measured using LPPAI scoring in a patient treated with the JAK inhibitor (JAKi) tofacitinib. This patient had previously failed monthly intralesional triamcinolone, topical steroids, doxycycline, and hydroxychloroquine (89). Similarly, AA patients have successfully been treated with JAKi (90–94). However, it is important to note that, like vitiligo, alopecia recurs upon cessation of JAKi (40). The known recurrence of AA and FFA, the confirmed upregulation of CD103+CD69+ TRM cells, and the reported clinical significance of JAKi support the crucial role of TRM cells in disease pathogenesis (50). A new clinical trial for a selective and simultaneous inhibitor of cytokines IL-2, IL-9, and IL-15 is enrolling for alopecia, which may have an impact on TRM (**Table S1**).

## Conclusion

The complex roles of TRM cells in autoimmune skin diseases are just beginning to be understood. However, it is clear that TRM play significant roles in disease reactivation and flares, and must be targeted if we hope to achieve long-lasting and durable treatment options for patients. The tremendous strides in the last ten years in understanding the pathophysiology and treatment of autoimmune skin disease is incredibly exciting, emphasizing not only how much we still have to learn about these diseases, but also the enormous impact it will have on patient survival and quality of life.

## Author Contributions

GR: original draft and figure drafts. JR and GR: literature search. GR and JR: digital artwork generation. JR and JH: editing. All authors contributed to the article and approved the submitted version. 

## Funding

GR was supported by the UMass Research & Curriculum Exploration program. JR is supported by a Career Development Award from the Dermatology Foundation, a Target Identification in Lupus Award from the Lupus Research Alliance and a Concept Award from the US Department of Defense Lupus Research Program #LR190030. JH is supported by NIH R01 AR069114, R61 AR070302, the Hartford Foundation, and a Calder Research Scholar Award from the American Skin Association. The funders had no role in study design, data collection and analysis, decision to publish, or preparation of the manuscript.

## Disclaimer

The views expressed in this article are those of the authors and may not reflect the official policy or position of the Department of the Army, Department of Defense, or the U.S. Government.

## Conflict of Interest

JH is the founder of Villaris Therapeutics and NIRA Biosciences. JH and JR are inventors on patent application #62489191, “Diagnosis and Treatment of Vitiligo” which covers targeting IL-15 and TRM for the treatment of vitiligo; and on patent application #15/851,651, “Anti-human CXCR3 antibodies for the Treatment of Vitiligo” which covers targeting CXCR3 for the treatment of vitiligo. JR is a reviewer for Frontiers Immunology journal.
